# The Protective Effect of Zinc Supplementation Against Oxidative Stress and Oxidative Modifications of Cellular Macromolecules in the Mandibular Bone of Rats Exposed to Cadmium

**DOI:** 10.3390/antiox14121480

**Published:** 2025-12-10

**Authors:** Kamil Bijowski, Ewa Dąbrowska, Małgorzata M. Brzóska, Joanna Rogalska, Karolina Orywal, Zofia N. Dąbrowska, Jan Borys

**Affiliations:** 1 Department of Maxillofacial and Plastic Surgery, Medical University of Bialystok, M. Skłodowskiej-Curie 24A Street, 15-276 Bialystok, Poland; 2 Independent Gerostomatology Laboratory, Medical University of Bialystok, Akademicka 3 Street, 15-267 Bialystok, Poland; 3 Department of Toxicology, Medical University of Bialystok, A. Mickiewicza 2C Street, 15-222 Bialystok, Poland; malgorzata.brzoska@umb.edu.pl (M.M.B.);; 4 Department of Biochemical Diagnostics, Medical University of Bialystok, J. Waszyngtona 15A Street, 15-269 Bialystok, Poland; karolina.orywal@umb.edu.pl; 5 Department of Radiology, Medical University of Bialystok, M. Skłodowskiej-Curie 24A Street, 15-276 Bialystok, Poland

**Keywords:** antioxidative enzymes, cadmium, lipid peroxides, mandibular bone, oxidised guanine metabolites, protection, protein carbonyl groups, total antioxidative status, total oxidative status, zinc

## Abstract

In a rat model simulating moderate and relatively high human exposure to cadmium (Cd; 5 and 50 mg/L, respectively, for 12 months), it was examined whether zinc (Zn) supplementation (30 and 60 mg/L, increasing the daily intake of this element by 71% and 146%, respectively) could protect against this xenobiotic-caused disruption of the oxidative–reductive balance in the mandibular bone tissue and the subsequent oxidative damage to nucleic acids, proteins, and lipids. The exposure to Cd weakened the enzymatic antioxidative barrier (superoxide dismutase (SOD), catalase (CAT), and glutathione peroxidase (GPx)) and decreased the total antioxidative status (TAS) of this tissue. The treatment with Cd also increased the concentration of hydrogen peroxide (H_2_O_2_) and the total oxidative status (TOS) of the mandibular bone tissue, leading to the development of oxidative stress, as indicated by an elevated value of the oxidative stress index (OSI), and oxidative damage to nucleic acids, lipids, and proteins. Zn supplementation at 30 and 60 mg/L during Cd exposure at 5 and 50 mg/L effectively protected against the accumulation of this toxic heavy metal in mandibular bone tissue and prevented oxidative stress and oxidative modifications of nucleic acids, proteins, and lipids. In conclusion, increasing Zn intake by at least 71% during chronic oral exposure to Cd may prevent oxidative–antioxidative imbalance and the development of oxidative stress, thereby safeguarding cellular macromolecules in the mandibular bone tissue from oxidative damage. These findings highlight the potential role of Cd exposure in the aetiology of mandibular bone damage and provide the first evidence that Zn supplementation may represent an effective intervention to alleviate adverse impact of long-term oral exposure to Cd on mandibular bone.

## 1. Introduction

Cadmium (Cd) is a toxic heavy metal that is entirely unnecessary for living organisms; nonetheless, it is detected in their bodies owing to its pervasive presence in the environment [1,2,3,4,5,6]. Besides dietary sources, habitual smoking of tobacco products constitutes the primary means of exposure of the general population to this deleterious element [1,7,8,9]. Because Cd accumulates in the tobacco plant (*Nicotiana tabacum*), a cigarette contains approximately 0.5–1 μg of this element [3,8]. Consequently, Cd concentrations in the blood, tissues, and urine of smokers are higher than those in nonsmokers [7,9]. Given Cd’s high accumulation in the human body and its capacity to induce adverse effects even at relatively low levels of exposure, it remains a significant environmental risk to public health. Chronic environmental and occupational exposure to this trace element poses a threat to various organs and systems, including the skeletal system [1,3,4,10,11,12,13,14,15,16]. Epidemiological research has demonstrated that low to moderate exposure to Cd, currently occurring in both industrialised and developing nations, constitutes a significant risk factor for skeletal injury, mainly osteoporosis [10,11,12,13,16]. Such exposure is associated with a reduction in bone mass and an increased risk of osteoporosis and fractures, predominantly in women (notably postmenopausal), who exhibit greater susceptibility to the toxic effects of Cd compared to men [10,11,13]. Moreover, exposure to this heavy metal may lead to its accumulation in the mandible and induce toxic effects within the teeth, thereby contributing to the progression of alterations associated with periodontal diseases [17,18,19,20]. Although the osteotoxic effects of this element have been extensively studied and documented in both humans [10,11,12,13,14,15,16] and experimental animals [21,22,23,24,25,26], its impact on mandibular bone tissue remains insufficiently understood.

It is well-established that the mechanisms underlying Cd osteotoxicity encompass both its direct and indirect effects on bone tissue [18,20,21,23,24,25,27]. The primary mechanism is believed to involve the direct influence of this xenobiotic on bone cells, including osteoblasts, osteoclasts, and osteocytes, thereby affecting their differentiation and metabolic activity [20]. Cd predominantly promotes bone resorption through the activation of osteoclasts and inhibits osteoblast activation. It reduces the expression of markers of osteoblast differentiation (such as Runx2 and osteocalcin) and of bone extracellular matrix proteins (type I collagen), and inhibits alkaline phosphatase activity, a zinc (Zn)-dependent enzyme essential for bone mineralisation [20,27,28]. Moreover, this toxic element impairs the differentiation of bone marrow mesenchymal stem cells into osteoblasts and induces their apoptosis [20,22]. The indirect effects predominantly relate to deficiencies in essential minerals, such as calcium and Zn, resulting from reduced gastrointestinal absorption and increased urinary excretion attributable to kidney damage [11]. The indirect pathway also includes reduced synthesis of the active vitamin D_3_ metabolite, 1,25-dihydroxyvitamin D_3_ (1,25(OH)_2_D_3_), in the kidneys, leading to decreased intestinal absorption of calcium and phosphorus [24].

One of the principal mechanisms by which Cd induces toxic effects in cells, including bone cells, is the induction of oxidative stress [3,4,23,25,29,30,31,32]. Although this heavy metal does not directly generate reactive oxygen species (ROS), it promotes a pro-oxidative state within cells through indirect mechanisms. These pathways involve the inhibition of the activity of enzymatic antioxidants, reduction in the concentration of non-enzymatic antioxidants, and the release of metal ions with high redox activity, predominantly iron(II) (Fe^2+^) and copper(I) (Cu^+^), from their binding sites, such as iron-sulphur proteins of the respiratory chain, ceruloplasmin, and ferritin [3,5,6]. Furthermore, the role of Cd in inducing oxidative stress involves disrupting electron flow through complex III (cytochrome bc1) in the mitochondrial electron transport chain [3,5,6].

Given that current environmental exposure to Cd poses health risks [10,11,13,14,15,16], researchers are investigating methods to mitigate its adverse effects. Owing to the pro-oxidative nature of Cd and the role of oxidative stress in its toxicity mechanisms, substances with antioxidative properties, including Zn [33,34,35,36,37,38], appear to be effective in this regard. This essential element demonstrates antioxidative activity through various mechanisms. Firstly, as a component of superoxide dismutase (SOD), which catalyses the dismutation of superoxide anion radicals into hydrogen peroxide (H_2_O_2_), it prevents the formation of other toxic radicals and their derivatives, such as hydroxyl or peroxynitrite radicals, both within the cytoplasm and extracellular matrix [35,36]. Another mechanism of Zn’s antioxidative action involves its competition with transition metal ions, such as Fe^2+^ and Cu^+^, for binding sites in proteins, lipids, or deoxyribonucleic acid (DNA), which participate in radical-generating reactions (e.g., the Fenton or Haber-Weiss reactions). Zinc ions (Zn^2+^) occupying these sites counteract such reactions [35,36]. Zn also protects the sulfhydryl (-SH) groups of proteins from oxidation by forming chelates that inhibit structural alterations in these molecules [38]. Additionally, this element induces the biosynthesis of metallothionein (MT), which can scavenge hydroxyl radicals and possesses an antioxidative capacity approximately 300 times greater than that of reduced glutathione (GSH) [37,39]. Evidence demonstrates that administering Zn to experimental animals during Cd exposure reduces Cd accumulation in the body and alleviates many of its toxic effects, including bone tissue damage [23,24,29,30,40,41]. It has been observed that increased Zn intake during oral exposure to Cd may protect against the accumulation and toxic action of this heavy metal in oral cavity organs, such as the salivary glands [30]; however, its effect on mandibular bone has not yet been thoroughly investigated.

Although Cd is primarily delivered to tissues, including mandibular bone, via systemic circulation, tobacco smokers can experience higher local oral exposure to this toxic element due to its presence in the tobacco smoke [3,7,8,9,42]. Thus, the adverse effects of this toxic heavy metal on the mandibular bone in tobacco smokers may be more pronounced than those observed in other bones, such as long bones like the femur and tibia. Cd can disrupt bone tissue metabolism through oxidative stress, with the oxidative–reductive status of the bone directly influencing the activity of these processes [23,25,27]. Since Zn possesses antioxidative properties [33,34,35,36,37,38] and has been reported to protect against Cd-induced oxidative stress in parenchymal organs and long bones [23,29,30,40,41], we hypothesised that increasing Zn supply would likewise mitigate stress and oxidative modifications of cellular macromolecules such as nucleic acids, proteins, and lipids within mandibular bone tissue. To evaluate this hypothesis, the present study measured the activities of antioxidative enzymes such as SOD, glutathione peroxidase (GPx), glutathione reductase (GR), and catalase (CAT), alongside total antioxidative status (TAS) and markers of oxidative status such as H_2_O_2_ and total oxidative status (TOS), as well as calculated the oxidative stress index (OSI). The total concentration of oxidised guanine metabolites (OGM), namely 8-hydroxyguanosine (8-OHG), 8-hydroxy-2′-deoxyguanosine (8-OHdG), and 8-hydroxyguanine, was determined as a biomarker of oxidative damage to DNA and ribonucleic acid (RNA). Furthermore, protein carbonyl groups (PC), serving as indicators of oxidative protein damage, and lipid peroxides (LPO), indicative of lipid peroxidation, were quantified in mandibular bone tissue. Cd and Zn concentrations within the mandibular bone were also measured, and their relationships with indices of the oxidative–reductive balance and oxidative modifications of cellular macromolecules were examined. To the best of our knowledge, no prior study of this kind has been conducted. Recognising that the oxidative–reductive status of bone tissue influences its turnover, density, and biomechanical properties [23,24,25], demonstrating in the current study that exposure to Cd can induce oxidative stress within the mandibular bone will suggest that this heavy metal may contribute to the propagation of mandibular bone damage. Moreover, establishing that Zn supplementation can protect against Cd-induced oxidative stress and its associated effects in the mandibular bone will imply that increasing Zn intake may serve as a protective measure against the deleterious effects of this toxic heavy metal on mandibular bone. Thus, conducting the present study was deemed necessary.

## 2. Materials and Methods

### 2.1. Animals and Experimental Design

The mandibular bones of rats utilised in the present study were obtained during an experiment authorised (approval No. 2004/03, 25 February 2004) by the Local Ethical Committee for Animal Experiments in Bialystok (Poland). All procedures involving the use of experimental animals were conducted in accordance with the ethical principles outlined in the International Guide for the Use of Animals in Biomedical Research and institutional guidelines.

The study involved 72 adult male Wistar rats (Hannover Wistar rats bred in accordance with the Charles River International Genetic Standardisation Program–Crl: WI (Han)), procured from a licenced Laboratory Animal House in Brwinów (Poland). Before the commencement of the study, the animals were acclimatised to the experimental conditions. No health abnormalities were detected during the acclimation period; consequently, all animals were included in the study. At the outset of the experiment, the rats were 10 weeks old and had a mean body weight of 220 ± 9.96 g (mean ± standard error of the mean (SE)). The experiment spanned 12 months. During this period, the rats were housed in stainless steel cages (four animals per cage) within a room maintained under standard husbandry conditions (relative humidity of 50 ± 10%, temperature of 22 ± 2 °C, and a 12-h light/dark cycle). The animals had unrestricted access to drinking water and a commercial standard dry (pelletised) rodent diet, LSM (Agropol; Motycz, Poland), formulated to contain 1.11% calcium, 0.72% phosphorus (P), vitamin D_3_ at a concentration of 1 IU/g, and Zn at 48 µg/g (manufacturer’s data). This diet fully met the animals’ requirements for all essential nutrients and contained only trace amounts of Cd (0.098 µg/g) [24].

The rats were randomly divided into nine groups, each consisting of eight subjects. The experimental design was as follows:

The control group—consisted of rats that were supplied with drinking water and the LSM standard chow, both free of pollutants, throughout the duration of the experiment.
The Zn30 group and the Zn60 group—consisted of animals that were administered an aqueous solution of zinc chloride (ZnCl_2_) at Zn concentrations of 30 and 60 mg/L, respectively, as their sole source of drinking fluid, along with the LSM standard chow
The Cd5 group and the Cd50 group—the rats were given, as the only drinking fluid, an aqueous solution of cadmium chloride (CdCl_2_) at Cd concentrations of 5 or 50 mg/L, respectively, and the LSM standard chow
The Cd5 + Zn30 group and the Cd5 + Zn60 group—the animals received an aqueous solution of CdCl_2_ and ZnCl_2_ containing Cd at the concentration of 5 mg/L and Zn at the concentration of 30 or 60 mg/L, respectively, as the only drinking fluid, along with the LSM standard chow
The Cd50 + Zn30 group and the Cd50 + Zn60 group—the animals received an aqueous solution of CdCl_2_ and ZnCl_2_ containing Cd at the concentration of 50 mg/L and Zn at the concentration of 30 or 60 mg/L, respectively, as the sole drinking fluid, and the LSM standard chow.

Redistilled water was selected as the drinking fluid for the control group and for preparing the aqueous solutions of CdCl_2_ and/or ZnCl_2_ to eliminate potential contaminants present in tap water, including Cd. Moreover, the use of redistilled water minimised potential interactions between the administered elements and minerals such as calcium, magnesium, zinc, and iron, whose concentrations in tap water may vary with water hardness and potentially influence the study outcomes.

The administration of drinking water containing Cd at concentrations of 5 and 50 mg/L to rats reflected moderate and relatively high human exposure to this xenobiotic, respectively. The daily intake of Cd in rats given this element at these concentrations (both separately and with Zn) ranged from 0.163 to 0.753 mg/kg body weight (b.w.) and from 1.740 to 4.440 mg/kg b.w., respectively (Table 1), with averages of 0.340 ± 0.026 mg/kg b.w. (mean ± SE) and 2.498 ± 0.093 mg/kg b.w., respectively [24]. Cd concentration in the blood and urine (key markers of exposure) of the animals was 1.223–2.121 μg/L and 0.0078–0.0262 μg/24 h, respectively, in the Cd5 group, and 14.97–17.20 μg/L and 0.2027–0.3647 μg/24 h, respectively, in the Cd50 group (Table S1) [24]. The Cd concentrations observed in the Cd5 group fall within the range reported in humans moderately exposed to this toxic element, both environmentally and occupationally, including tobacco smokers (Table S2). Furthermore, to examine the dose-effect relationship and the potential benefits of Zn at relatively high Cd exposure, the concentration of 50 mg/L was also tested.

The daily intake of Zn in the groups receiving this element at the concentrations of 30 and 60 mg/L, both alone and during exposure to Cd, was similar, regardless of whether it was administered alone or with Cd. It ranged from 0.980 to 3.980 mg/kg b.w. and from 2.020 to 7.710 mg/kg b.w., respectively, with a mean ± SE of 1.904 ± 0.123 mg/kg b.w. and 3.699 ± 0.213 mg/kg b.w., respectively (Table 1) [24]. Adding ZnCl_2_ to drinking water at 30 and 60 mg/L increased the daily intake of this essential element in rats by 71% and 146%, respectively, compared with intake from the standard diet [24]. Zn concentration in the serum and urine of the rats in the respective experimental groups has already been reported (Table S3) [24].

Throughout the study, no differences in daily water or food intake were observed. All animals gained body weight, and by the end of the study, there were no differences in body weight between the experimental groups [24]. The animals tolerated the daily doses of Cd and/or Zn well. Daily clinical observations throughout the experiment showed no noticeable health abnormalities in these animals.

At the end of the experiment, the animals were subjected to deep barbiturate anaesthesia via intraperitoneal injection of Vetbutal at a dose of 30 mg/kg b.w. (Biowet, Pulawy, Poland). Blood was collected from the heart, and various organs, including mandibular bones, were dissected [24]. After being cleaned of soft tissues, the mandibular bones were weighed immediately, protected from desiccation, and stored at −80 °C until used in the present study.

### 2.2. Preparation of the Mandibular Bone for Testing

**Figure 1:**
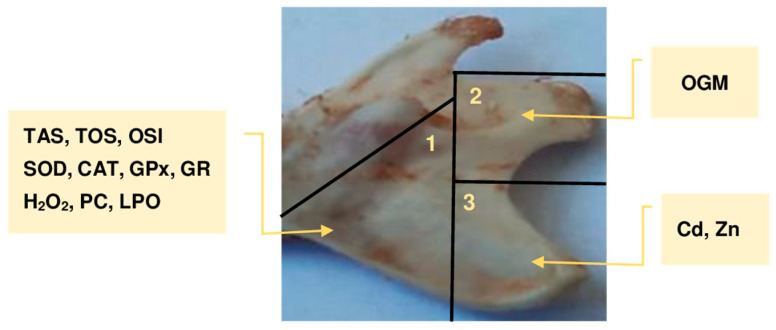
A representative rat’s mandibular bone and the method used to section it into three parts for testing, along with the scope of the assessments performed on each part. CAT, catalase; Cd, cadmium; GPx, glutathione peroxidase; GR, glutathione reductase; H_2_O_2_, hydrogen peroxide; LPO, lipid peroxides; OGM, oxidised guanine metabolites; OSI, oxidative stress index; PC, protein carbonyls; SOD, superoxide dismutase; TAS, total antioxidative status; TOS, total oxidative status; Zn, zinc.

After thawing the mandibular bones to ambient temperature, they were visually inspected and their weight reassessed. All bones appeared fresh, with no signs of deterioration since freezing, and their weights were consistent with those measured immediately after collection. Next, the mandibular bones were sectioned into three parts as illustrated in Figure 1. The first part (0.077–0.227 g) was subjected to the determination of markers of oxidative–reductive status, except for OGM (Figure 1). From this tissue, two 10% homogenates were prepared in a cold phosphate buffer (pH 7.4) using a knife homogeniser (Ultra-Turrax T25 from IKA, Staufen, Germany). To prevent autooxidation of the tested material, 0.5 M butylhydroxytoluene (Sigma-Aldrich GmbH, Steinheim, Germany) in acetonitrile (0.01 mL per 1 mL of homogenate) (Merck, Darmstadt, Germany) was added to the samples. Moreover, to the portion of the bone tissue subjected to CAT determination [43], 1% Triton X-100 (Merck, Darmstadt, Germany) was added before homogenisation (0.1 mL per 1 mL of homogenate). Homogenates prepared without Triton X-100 were divided into two portions: one designated for TAS, LPO, H_2_O_2_, and TOS assays, and the other for SOD, GPx, GR, and PC. Immediately after preparation, the samples for the determination of CAT, TAS, LPO, H_2_O_2_, and TOS were centrifuged at 700× *g* for 20 min (at 4 °C), while those for SOD, GPx, GR, and PC assay were centrifuged at 20,000× *g* for 30 min (at 4 °C) [44]. After centrifugation, the received aliquots were separated immediately and kept frozen (−80 °C) until the scheduled assays were performed.

The second segment of the mandibular bone, with a precise weight of 0.1 g each (Figure 1), was employed to quantify OGM (the aggregate concentrations of 8-OHG, 8-OHdG, and 8-hydroxyguanine) as an indicator of oxidative damage to DNA/RNA. The extraction of genetic material from the mandibular bone adhered to the protocol outlined in the Nuclear Extraction Kit (No. 10009277) supplied by Cayman Chemical Company (Ann Arbor, MI, USA). A volume of 0.3 mL of ice-cold buffer containing dithiothreitol and nonidet P-40 (3 μL of 1 M dithiothreitol and 3 μL of 10% nonidet P-40) was utilised to prepare the bone homogenate from 0.1 g of the mandibular bone tissue, employing an Ultra-Turrax T25 homogeniser from IKA (Staufen, Germany). Following purification, conducted diligently in accordance with the instructions provided in the kit manual, the homogenate was centrifuged at 14,000× *g* for 30 s at 4 °C. The supernatant was then carefully separated.

The values of all variables measured in the aliquots of mandibular bone tissue homogenates were adjusted for total protein concentration. Protein quantification was conducted utilising a BioMaxima kit (No. 1-055-0200; Lublin, Poland), with an assay precision indicated by a coefficient of variation (CV) < 6%. The spectrophotometers Epoch (BioTek Instruments, Inc., Winooski, VT, USA) and Specord 50 Plus (Analytik Jena, Jena, Germany) were employed to measure the specified variables in the aliquots of mandibular bone homogenates.

In the third section of the mandibular bone (Figure 1), the concentrations of Zn and Cd were determined. The bone samples were subjected to wet digestion using concentrated, high-purity nitric acid (65%; Merck, Darmstadt, Germany) and hydrochloric acid (30%; Merck), employing a closed-loop microwave system (Uniclever II microwave mineraliser, Plazmatronika, Wrocław, Poland). The mineralisation process involved four stages at varying temperatures (1st—170 °C, 2nd—190 °C, 3rd—210 °C, and 4th—50 °C) and pressures (1st—20 atm, 2nd—30 atm, 3rd—40 atm, and 4th—40 atm), with durations of 10, 10, 10, and 18 min, respectively.

### 2.3. Determination of TAS and TOS

TAS and TOS measurements were performed using the ImAnOx (TAS) Kit (No. KC5200) and PerOx (TOS) Kit (No. KC5100), both supplied by Immundiagnostik AG (Bensheim, Germany). The TAS values obtained in the control samples included with the kit were 189.5 ± 7.50 and 230.0 ± 13.00 µmol/L (mean ± SE, n = 2), which fell within the manufacturer-specified ranges (170–230 and 195–263 µmol/L, respectively). The method’s precision, expressed as the CV, was <5%. The TOS values measured in the control samples included with the kit were 173.4 ± 3.15 and 661.7 ± 28.70 µmol/L (mean ± SE, n = 2) and were within the manufacturer-specified ranges (116–194 and 423–705 µmol/L, respectively). The CV of the assay was <3%. The OSI value was calculated as the ratio of TOS to TAS.

### 2.4. Determination of GPx, GR, SOD, and CAT Activities

The activities of GPx and GR were quantified utilising the kinetic method with the Bioxytech GPx-340 TM kit (No. 21017) and Bioxytech GR-340 TM kit (No. 21018), respectively, from the Oxis Research division of AOXRE LLC (Burlingame, CA, USA). The CV of the measurements was <4% and <5%, respectively.

SOD activity was measured spectrophotometrically using the Superoxide Dismutase Assay Kit (No. 706002) supplied by Cayman Chemical Company (Ann Arbor, MI, USA). The CV of the method was <4%.

CAT activity was determined employing a spectrophotometric technique as described by Aebi [43]. The assay measures the rate of H_2_O_2_ decomposition by this enzyme, with measurements taken at 240 nm. The CV of the assay was <3%.

### 2.5. Determination of H_2_O_2_ Concentration

The concentration of H_2_O_2_ was measured using the Bioxytech^®^ H_2_O_2_-560^TM^ kit (No. 21024) from OxisResearch^TM^–Aoxre LLC (Burlingame, CA, USA). The CV for the method was <2%.

### 2.6. Determination of OGM, PC, and LPO Concentrations

The concentrations of OGM and PC were determined using the DNA/RNA Oxidative Damage (High Sensitivity) ELISA Kit (No. 589320) and the Protein Carbonyl Colorimetric Assay Kit (No. 10005020), respectively, from Cayman Chemical Company (Ann Arbor, MI, USA). The CVs for these assays were <2% and <4%, respectively. LPO concentration was determined with the Bioxytech^®^LPO-586^TM^ kit (No. 21012) from OxisResearch^TM^–Aoxre LLC (Burlingame, CA, USA). The method’s CV was <5%.

### 2.7. Determination of Cd and Zn Concentrations

The concentrations of Cd and Zn in the solutions of wet-digested mandibular bone were measured using an acetylene-air flame (for Zn) and electrothermal (for Cd) atomic absorption spectrometry with Zeeman background correction (Z-5000 instrument, Hitachi, Japan). The procedures were performed according to the manufacturer’s recommendations. Each measurement was performed in triplicate. Cd and Zn concentrations in bone samples were expressed as μg/g.

Quality control of these measurements was carried out by analysing the Standard Reference Material 1400 Bone Ash (No. 1400; National Institute of Standards and Technology, Gaithersburg, MD, USA). The certified material was subjected to the same pretreatment and analysis procedure as the mandibular bone samples. The concentrations of Cd and Zn measured in the reference material analysed simultaneously were 0.033 ± 0.002 μg/g and 176.7 ± 3.5 μg/g (mean ± SE, n = 2), respectively, and were within the range of the manufacturer’s specified values (0.03 ± 0.005 μg/g and 181 ± 3 μg/g, respectively).

### 2.8. Statistical Analysis

The results were analysed statistically using Statistica 13 (StatSoft; Tulsa, OK, USA). Since the Shapiro-Wilk test indicated that the data did not follow a normal distribution, the statistical significance of the differences between the experimental groups was evaluated using the non-parametric Kruskal-Wallis test (*p* < 0.05). The results were presented as median, 25–75% confidence interval, and minimum and maximum values for eight rats in each group. The effect size, which quantifies the strength of the difference between the tested groups, was calculated as eta squared (η^2^) with categories designated as large (η^2^ ≥ 0.14), medium (0.01 < η^2^ < 0.14), and weak (η^2^ ≤ 0.01). Spearman’s correlation analysis was performed to estimate the relationships between the concentrations of Cd and Zn and the assessed indices of oxidative–reductive status of the mandibular bone tissue, and to determine the mutual dependencies among the assayed indices of oxidative–reductive status. Moreover, dependencies between the main indices of the oxidative–reductive status (TAS, TOS, and OSI) of the mandibular bone and the concentrations of Cd and Zn in the bone tissue determined in the present study and previously reported by us, concentrations of these elements in the blood/serum and their 24-h urinary excretion [24] were evaluated. In the case of the existence of a relationship between two parameters (*p* < 0.05), the degree of correlation was also estimated. The correlation was very strong for a coefficient of correlation ׀r ׀≥ 0.9, strong for 0.9 > |r| ≥ 0.7, moderate for 0.7 > |r| ≥ 0.4, and weak for 0.4 > |r| ≥ 0.2.

## 3. Results

### 3.1. Effects of Cd and/or Zn on TAS, TOS, and OSI of the Mandibular Bone Tissue

**Figure 2:**
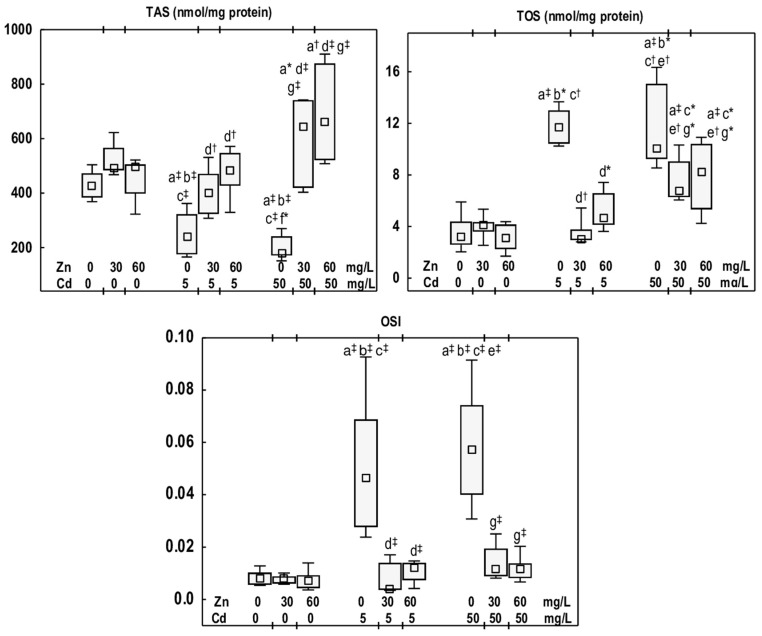
Total antioxidative status (TAS), total oxidative status (TOS), and oxidative stress index (OSI) in the mandibular bone tissue of male rats after 12-month treatment with cadmium (Cd) (5 or 50 mg/L) and/or zinc (Zn) (30 or 60 mg/L) in drinking water, along with the control group. Data are presented as medians with 25–75% confidence intervals and minimum and maximum values for eight rats in each group. Statistically significant differences (non-parametric signed-rank Kruskal–Wallis test): a vs. the control group, b vs. the Zn30 group, c vs. the Zn60 group, d vs. the Cd5 group, e vs. the Cd5 + Zn30 group, f vs. the Cd5 + Zn60 group, and g vs. the Cd50 group, where * *p* < 0.05, ^†^ *p* < 0.01, and ^‡^ *p* < 0.001. The effect size (η^2^) for the differences in TAS, TOS, and OSI between the experimental groups was large (0.657, 0.753, and 0.581, respectively). Detailed data are available in Table S4.

TAS, TOS, and OSI values in animals given Zn at 30 or 60 mg/L (the Zn30 and Zn60 groups) were comparable to those observed in the control group (Figure 2).

The exposure of rats to Cd at the concentrations of 5 and 50 mg/L resulted in a reduction in TAS (by 43% and 58%, respectively) and an increase in TOS (3.6- and 3.1-fold, respectively), as well as an elevation in OSI (5.8- and 7.3-fold, respectively) (Figure 2). No differences were noted in TAS, TOS, and OSI between the Cd5 and Cd50 groups (Figure 2).

The animals supplemented with Zn at 30 and 60 mg/L during exposure to Cd at 5 mg/L (the Cd5 + Zn30 and Cd5 + Zn60 groups) showed higher TAS than the Cd5 group (by 66% and 100%, respectively). TOS and OSI were reduced (from 2.5- to 11-fold) compared with the Cd5 group, and did not differ from the control group (Figure 2). In the groups administered Zn at 30 or 60 mg/L during Cd exposure at 50 mg/L, TAS was higher than in the Cd50 group (3.6- and 3.7-fold, respectively) and the control group (by 51% and 54%, respectively). TOS in the Cd50 + Zn30 and Cd50 + Zn60 groups was reduced (by 33% and 18%, respectively) relative to the Cd50 group, yet was higher (2.1- and 2.5-fold, respectively) than in the control group (Figure 2). OSI in these groups was lower (5-fold and 4.9-fold, respectively) than in the Cd50 group and did not differ from the control group (Figure 2). There were no differences in TAS, TOS, and OSI between the Cd5 + Zn30 and Cd5 + Zn60 groups, nor between the Cd50 + Zn30 and Cd50 + Zn60 groups; however, TOS in the Cd50 + Zn30 group was higher (2.2-fold) compared to the Cd5 + Zn30 group (Figure 2).

### 3.2. Effects of Cd and/or Zn on the Enzymatic Antioxidative Defence of Mandibular Bone Tissue

**Figure 3:**
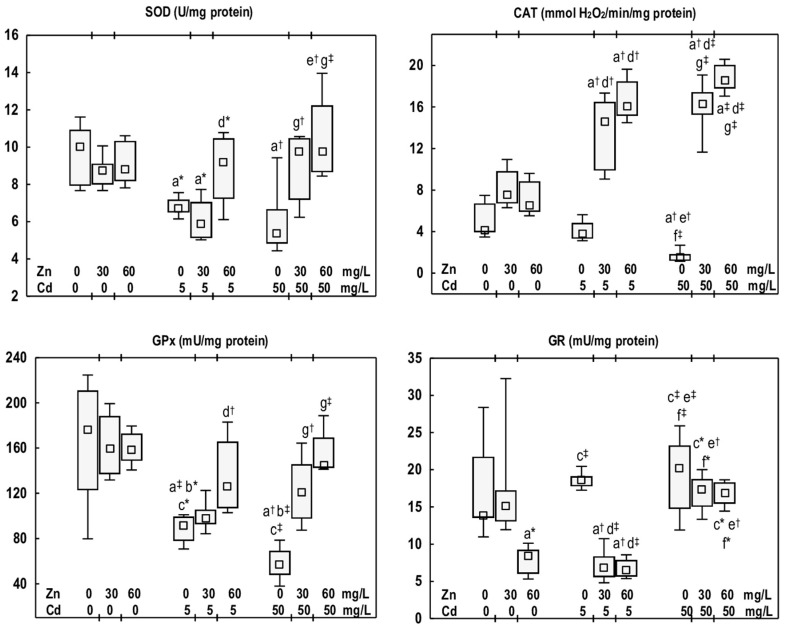
The activities of superoxide dismutase (SOD), catalase (CAT), glutathione peroxidase (GPx), and glutathione reductase (GR) in the mandibular bone tissue of male rats after 12-month treatment with cadmium (Cd) (5 or 50 mg/L) and/or zinc (Zn) (30 or 60 mg/L) in drinking water, along with the control group. Data are presented as medians with 25–75% confidence intervals, and minimum and maximum values, for eight rats in each experimental group. Statistically significant differences (non-parametric signed-rank Kruskal–Wallis test): a vs. the control group, b vs. the Zn30 group, c vs. the Zn60 group, d vs. the Cd5 group, e vs. the Cd5 + Zn30 group, f vs. the Cd5 + Zn60 group, and g vs. the Cd50 group, where * *p* < 0.05, ^†^ *p* < 0.01, and ^‡^ *p* < 0.001. The effect size (η^2^) for the differences in the activities of SOD, CAT, GPx, and GR between the experimental groups was large (0.500, 0.890, 0.640, and 0.684, respectively). Detailed data are available in Table S5.

In the groups of rats administered Zn at 30 or 60 mg/L, the activities of SOD, CAT, GPx, and GR in the mandibular bone tissue were comparable to those in the control group, except for a decreased (by 38%) GR activity in the Zn60 group (Figure 3).

The exposure of rats to Cd at 5 or 50 mg/L resulted in a reduction in the activities of SOD (by 33% and 46%, respectively) and GPx (by 48% and 68%, respectively). The activity of CAT was diminished (by 63%) exclusively in the Cd50 group, whereas the activity of GR remained unaffected (Figure 3). The activities of antioxidative enzymes did not differ between the Cd5 and Cd50 groups (Figure 3).

The activity of SOD in the Cd5 + Zn60 group was elevated (by 37%) compared to the Cd5 group. In the Cd50 + Zn30 and Cd50 + Zn60 groups, this enzyme activity was increased (by 82% and 81%, respectively) relative to the Cd50 group, and exhibited no difference from the control group (Figure 3). CAT activity in rats supplemented with Zn at both levels of Cd exposure was markedly higher (3.8- and 4-fold in the Cd5 + Zn30 and Cd5 + Zn60 groups, respectively, and 11- and 12-fold in the Cd50 + Zn30 and Cd50 + Zn60 groups, respectively) compared to the respective groups treated solely with Cd. Furthermore, this enzyme activity in Zn-supplemented animals was elevated relative to the control group (from 3.6- to 4.5-fold, respectively) (Figure 3). GPx activity in the Cd5 + Zn30 and Cd5 + Zn60 groups remained comparable to that of the control group; notably, the Cd5 + Zn60 group demonstrated an increase (by 37%) over the Cd5 group. In animals receiving Zn during the higher Cd exposure, this enzyme activity was increased (2-fold in the Cd50 + Zn30 group and 2.5-fold in the Cd50 + Zn60 group) relative to the Cd50 group, and it did not differ from the control group (Figure 3). GR activity in the rats supplemented with Zn during the exposure to Cd at 5 mg/L was reduced compared with the Cd5 group (by 63% and 65%, respectively) and the control group (by 50% and 52%, respectively). However, Zn supplementation during the higher Cd exposure (50 mg/L) did not influence GR activity (Figure 3). No differences were noted in the activities of antioxidative enzymes between the Cd5 + Zn30 and Cd5 + Zn60 groups, nor between the Cd50 + Zn30 and Cd50 + Zn60 groups. Nonetheless, GR activity in the Cd50 + Zn30 group was higher (by 2.5-fold) than in the Cd5 + Zn30 group (Figure 3).

### 3.3. H_2_O_2_ Concentration in the Mandibular Bone Tissue

**Figure 4:**
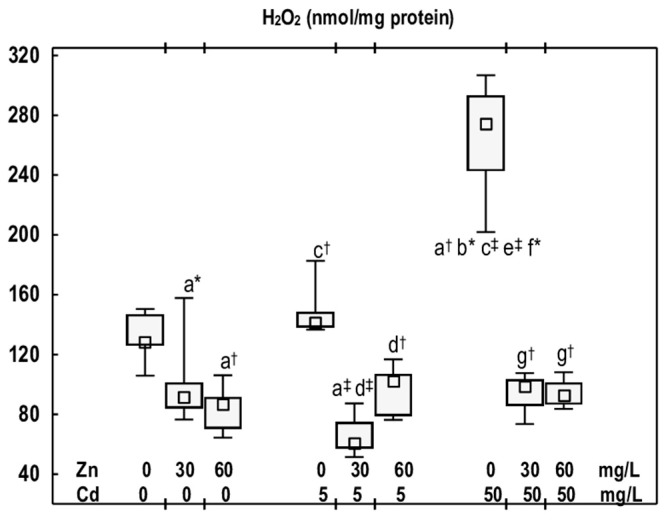
The concentration of hydrogen peroxide (H_2_O_2_) in the mandibular bone tissue of male rats after 12-month treatment with cadmium (Cd) (5 or 50 mg/L) and/or zinc (Zn) (30 or 60 mg/L) in drinking water, along with the control group. Data are presented as medians, 25–75% confidence intervals, and minimum and maximum values for eight rats in each experimental group. Statistically significant differences (non-parametric signed-rank Kruskal–Wallis test): a vs. the control group, b vs. the Zn30 group, c vs. the Zn60 group, d vs. the Cd5 group, e vs. the Cd5 + Zn30 group, f vs. the Cd5 + Zn60 group, and g vs. the Cd50 group, where * *p* < 0.05, ^†^ *p* < 0.01, and ^‡^ *p* < 0.001. The effect size (η^2^) for the differences in H_2_O_2_ concentration between the experimental groups was large (0.747). Detailed data are available in Table S6.

In the Zn30 and Zn60 groups, H_2_O_2_ concentration in the mandibular bone tissue was lower (by 29% and 32%, respectively) than in the control group (Figure 4).

In the animals that received Cd at 5 mg/L for 12 months, the concentration of H_2_O_2_ remained comparable to that of the control group; however, at the higher exposure, it increased (2-fold) (Figure 4). Nonetheless, no difference in H_2_O_2_ concentration was observed between the Cd5 and Cd50 groups (Figure 4).

The concentration of H_2_O_2_ in the mandibular bone tissue of rats supplemented with Zn at both levels of Cd exposure was reduced (by 28–66%) compared to the respective groups treated with Cd alone, and it remained within the range of the control group (Figure 4). No difference was observed in the concentration of this compound between the Cd5 + Zn30 and Cd5 + Zn60 groups, nor between the Cd50 + Zn30 and Cd50 + Zn60 groups (Figure 4).

### 3.4. Effects of Cd and/or Zn on Markers of Oxidative Modifications of Cellular Macromolecules in the Mandibular Bone Tissue

**Figure 5:**
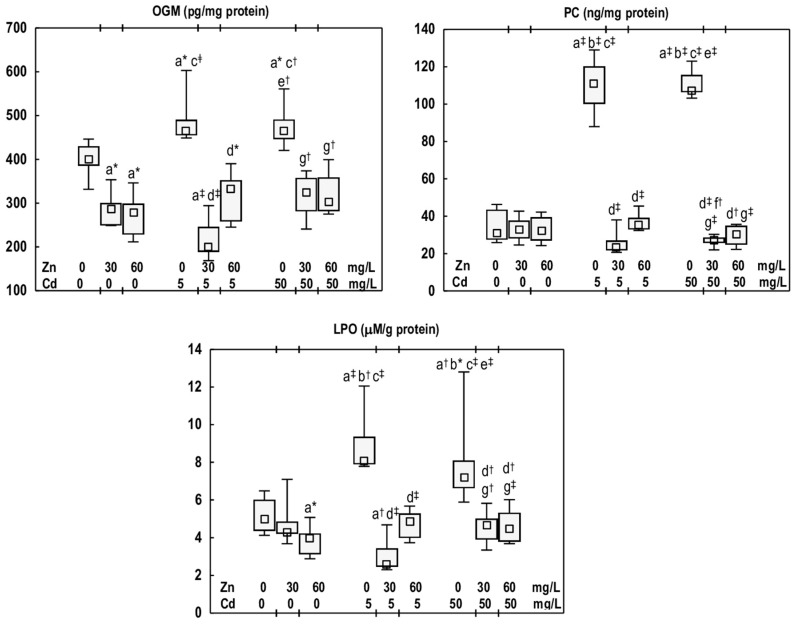
The concentrations of oxidised guanine metabolites (OGM), protein carbonyls (PC), and lipid peroxides (LPO) in the mandibular bone tissue of male rats after 12-month treatment with cadmium (Cd) (5 or 50 mg/L) and/or zinc (Zn) (30 or 60 mg/L) in drinking water, along with the control group. Data are presented as medians with 25–75% confidence intervals and minimum and maximum values for eight rats in each experimental group. Statistically significant differences (non-parametric signed-rank Kruskal–Wallis test): a vs. the control group, b vs. the Zn30 group, c vs. the Zn60 group, d vs. the Cd5 group, e vs. the Cd5 + Zn30 group, f vs. the Cd5 + Zn60 group, and g vs. the Cd50 group, where * *p* < 0.05, ^†^ *p* < 0.01, and ^‡^ *p* < 0.001. The effect size (η^2^) for the differences in the concentrations of OGM, PC, and LPO between the experimental groups was large (0.755, 0.631, and 0.646, respectively). Detailed data are available in Table S6.

In the groups of rats administered with Zn at 30 or 60 mg/L, the concentration of OGM in the mandibular bone tissue was lower (by 28% and 30%, respectively) than in the control group (Figure 5). The PC concentration in these animals remained consistent with that observed in the control group, while LPO concentration was lower (19%) in the Zn60 group (Figure 5).

Cd exposure (the Cd5 and Cd50 groups) resulted in an increase in the concentrations of OGM (by 16% in both groups), PC (3.6- and 3.4-fold, respectively), and LPO (by 63% and 45%, respectively) in the mandibular bone (Figure 5). No differences were noted in the values of these biomarkers between the Cd5 and Cd50 groups (Figure 5).

The concentration of OGM in the Cd5 + Zn30 and Cd5 + Zn60 groups was lower (by 57% and 28%, respectively) compared to the Cd5 group. In the case of the Cd5 + Zn30 group, the reduction was also observed (by 50%) relative to the control group (Figure 5). Among the animals supplemented with Zn at the higher level of Cd exposure (the Cd50 + Zn30 and Cd50 + Zn60 groups), the concentration of this marker of nucleic acids damage was diminished (by 30% and 35%, respectively) compared to the Cd50 group, and it did not differ from that noted in the control group (Figure 5). The PC concentration in the mandibular bone of rats supplemented with Zn and exposed to Cd at 5 and 50 mg/L was decreased (by 68–79%) compared with the respective groups treated solely with this xenobiotic, and was comparable to the control group (Figure 5). In the animals supplemented with Zn at both levels of Cd exposure, the concentration of LPO was reduced (by 35–68%) relative to the Cd5 or Cd50 groups, and did not differ from that of the control group (Figure 5). No differences in the concentrations of the examined markers of oxidative damage to the nucleic acids, proteins, and lipids were noted between the Cd5 + Zn30 and Cd5 + Zn60 groups and the Cd50 + Zn30 and Cd50 + Zn60 groups (Figure 5).

### 3.5. Cd and Zn Concentrations in the Mandibular Bone Tissue

**Figure 6:**
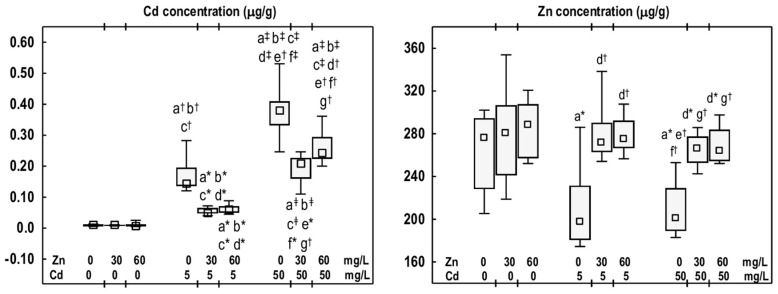
The concentrations of cadmium (Cd) and zinc (Zn) in the mandibular bone tissue of male rats after 12-month treatment with Cd (5 or 50 mg/L) and/or Zn (30 or 60 Zn/L) in drinking water, along with the control group. Data are presented as medians with 25–75% confidence intervals and minimum and maximum values for eight rats in each experimental group. Statistically significant differences (non-parametric signed-rank Kruskal–Wallis test): a vs. the control group, b vs. the Zn30 group, c vs. the Zn60 group, d vs. the Cd5 group, e vs. the Cd5 + Zn30 group, f vs. the Cd5 + Zn60 group, and g vs. the Cd50 group, where * *p* < 0.05, ^†^ *p* < 0.01, and ^‡^ *p* < 0.001. The effect size (η^2^) for the differences in the concentrations of Cd and Zn between the experimental groups was large (0.362 and 0.914, respectively). Detailed data are available in Table S7.

Cd concentration in the mandibular bone tissue in the Zn30 and Zn60 groups was comparable to that assayed in the control group (Figure 6). Exposure to Cd at 5 mg/L resulted in a 15-fold increase in the concentration of this toxic element in the mandibular bone. However, in the higher treatment group (the Cd50 group), a 40-fold increase was observed compared to the control group (Figure 6). The Cd concentration in the Cd50 group was 2.6 times higher than that in the Cd5 group. The animals supplemented with Zn at 30 or 60 mg/L during the exposure to Cd at 5 or 50 mg/L exhibited lower Cd concentrations (by 36–65%) than those in the corresponding groups treated solely with Cd, but higher (from 5- to 26-fold) than in the control group (Figure 6).

Zn concentration in the mandibular bone tissue of animals administered this element at 30 or 60 mg/L was comparable to the control group (Figure 6). Exposure to Cd alone (Cd5 and Cd50 groups) resulted in a reduction in Zn concentration (by 28% and 27%, respectively) (Figure 6). The animals receiving Zn at 30 or 60 mg/L during exposure to Cd at 5 or 50 mg/L, exhibited higher concentration of this bioelement than in the Cd5 group (by 37% and 39%, respectively) and the Cd50 group (by 33% and 31%, respectively). These concentrations were comparable to those recorded in the control group (Figure 6). No differences were observed in Cd and Zn concentrations between the Cd5 + Zn30 and Cd5 + Zn60 groups, as well as the Cd50 + Zn30 and Cd50 + Zn60 groups (Figure 6).

### 3.6. Dependencies Among Variables Measured in the Mandibular Bone

Spearman’s correlation analysis revealed a positive association between Zn concentration in mandibular bone and the activities of SOD, CAT, and GPx, as well as TAS. Conversely, GR activity, TOS, OSI, and the concentrations of OGM, PC, LPO, and H_2_O_2_ exhibited negative correlations with Zn concentration (Table 2). Furthermore, a negative correlation was observed between Cd concentration in the mandibular bone and SOD and GPx activities. However, TOS, OSI, GR activity, and the concentrations of OGM, PC, LPO, and H_2_O_2,_ positively correlated with Cd concentration (Table 2). The mandibular bone tissue concentrations of Zn and Cd correlated negatively (r = −0.340, *p* < 0.01).

Furthermore, there were also numerous mutual dependencies observed among the measured indicators of oxidative–reductive status in the mandibular bone. TAS correlated positively with the activities of SOD, CAT, and GPx, and negatively with OSI and the concentrations of OGM, PC, and H_2_O_2_. TOS exhibited a negative correlation with GPx activity and a positive correlation with GR activity, OSI, and the concentrations of OGM, PC, LPO, and H_2_O_2_ (Table 2).

### 3.7. Dependencies Between TAS, TOS, OSI, and the Concentrations of Cd and Zn in the Mandibular Bone, and These Element Concentrations in the Blood/Serum and Their 24-h Urinary Excretion

The mandibular bone TOS and OSI showed a positive correlation with Cd concentration in the blood and the 24-h urinary excretion of Cd and Zn. A negative correlation was observed between the mandibular bone TOS and the serum Zn concentration. No dependency was identified between the bone TAS and the concentrations of Cd and Zn in the blood/serum, nor with their daily urinary excretion (Table 3).

A strong positive correlation was disclosed between Cd concentration in the mandibular bone and the concentration of this toxic element in the blood, as well as its 24-h urinary excretion. Additionally, Cd concentration in the mandibular bone exhibited a positive association with urinary Zn excretion. Conversely, a negative correlation was found between bone Cd and serum Zn concentrations (Table 3).

## 4. Discussion

The present study provides new data on Cd toxicity, suggesting that exposure to this heavy metal may contribute to the development of mandibular bone damage, and explores the potential protective effects of Zn supplementation against this action. A pivotal finding is that chronic exposure to Cd, representative of moderate human exposure, results in its distribution into mandibular bone, thereby disrupting redox balance and inducing oxidative stress. This process subsequently causes oxidative modifications of nucleic acids, lipids, and proteins. Of particular importance is the finding that increasing Zn intake by at least 71% during both moderate and relatively high Cd exposure confers protection against the retention of this toxic element in mandibular bone and inhibits the onset of oxidative stress and damage to cellular macromolecules. Furthermore, this study reinforces previous findings by our research team [23,24,29,30,40,41], indicating that Zn supplementation constitutes an effective strategy for mitigating the adverse health effects associated with prolonged oral exposure to Cd. Notably, this study is the first to report on the protective effect of Zn against the detrimental impact of Cd on mandibular bone.

Browar et al. [17] documented a higher concentration of Cd in the alveolar process of the mandible (the superior, tooth-bearing segment of the mandible) in comparison to the basal bone. Furthermore, a positive correlation has been identified between the concentration of Cd in the mandibular alveolar process and the prevalence of periodontal diseases, indicating that the deleterious effects of this toxic element may stem from a direct osteotoxic effect on the alveolar process [17]. Consequently, given the relationship between the redox status of bone tissue and bone metabolism [23,25,45], it is imperative, when assessing the risks associated with exposure to pro-oxidants such as Cd and exploring potential mitigation strategies, to evaluate both the destructive influence of Cd and the protective role of Zn, an antioxidant, on the oxidative–reductive status of mandibular bone.

The present study demonstrates that during oral exposure to Cd, the heavy metal distributes into the mandibular bone in a dose-dependent manner. In the control group, as well as in the Zn30 and Zn60 groups, which were fed diet containing only trace amounts of Cd (0.098 µg/g [24]) and drank water without added CdCl_2_ (Cd concentration < 0.05 μg/L [24]), the concentration of this element in the mandibular bone was very low (0.0054–0.0251 μg/g). However, it significantly increased after 12 months of moderate to relatively high exposure to Cd, leading to severe disruption of the redox balance and oxidative stress, which elicited adverse outcomes, including oxidative modifications of nucleic acids, proteins, and lipids in the mandibular bone tissue.

Bone tissue possesses defence mechanisms designed to safeguard it against the harmful effects of ROS. The antioxidative barrier within bone comprises both enzymatic antioxidants, including SOD, CAT, GPx, and GR, as well as non-enzymatic counterparts such as GSH and MT. Among these, GPx is identified as the most potent enzymatic component of the antioxidative defence system in bone tissue. This enzyme facilitates the breakdown of lipid peroxides and, in conjunction with CAT, catalyses the reduction of H_2_O_2_ to molecular oxygen and water. SOD catalyses the dismutation of superoxide anion radicals into molecular oxygen and H_2_O_2_, whereas GR catalyses the reduction in oxidised glutathione (GSSG) to its reduced form (GSH) [36,46]. The decline in TAS within mandibular bone tissue of rats exposed to Cd at 5 and 50 mg/L, in conjunction with elevated H_2_O_2_ concentration, TOS, and OSI, suggests that the antioxidative defence mechanisms in this tissue have been compromised. These effects may be attributed to the attenuation of enzymatic defence mechanisms induced by this xenobiotic, as observed in the present study. The diminished activities of SOD, CAT, and GPx could result from Cd’s capacity to bind to sulfhydryl groups (-SH groups), disrupt the metabolism of essential bioelements, such as selenium (Se), Zn, copper (Cu), manganese (Mn), and iron (Fe), which are necessary for optimal enzymatic function, and displace these bioelements from their active sites [23,24,30,40,46]. Cd ions (Cd^2+^) can displace Se ions (Se^2+^) from GPx, iron(III) ions (Fe^3+^) from CAT, and Zn^2+^, Cu^+^, and Mn(II) ions (Mn^2+^) from the active sites of SOD [24,40]. The observed decrease in SOD activity in the mandibular bone tissue may partly be attributed to a reduction in Zn concentration, as identified in this study at both levels of Cd exposure. A decrease in Zn concentration has already been reported in the femur of these rats (by 7% and 19% in the Cd5 and Cd50 groups, respectively) [24]. Considering previous findings on the antioxidative capacity of the femoral bone tissue of these animals [23], it is plausible that the Cd-induced decrease in TAS in mandibular bone may also be related to effects on non-enzymatic antioxidants.

The Cd-induced attenuation of the oxidative defence mechanisms observed in mandibular bone tissue in the current study compromised ROS detoxification, thereby increasing ROS levels. This was evidenced by increased H_2_O_2_ concentration and TOS, which disrupted the redox balance, leading to oxidative modifications of cellular macromolecules, such as nucleic acids and proteins, and to enhanced lipid peroxidation in the mandibular bone. Since oxidative stress underpins numerous pathological processes in tissues and organs, including those within the oral cavity [31,47,48], these findings underscore the toxic impact of Cd on the mandible and suggest that prolonged exposure to this toxic heavy metal may contribute to the development of periodontal diseases.

It is crucial to emphasise that, despite variations in exposure intensity and Cd concentration within mandibular bone tissue, the pro-oxidative effects of this xenobiotic were observed to be dose-independent. Although the median values for specific parameters (CAT, GPx, and H_2_O_2_) in the Cd5 and Cd50 groups clearly differed, the difference did not reach statistical significance. This suggests that the effects of Cd are primarily mediated by indirect mechanisms rather than the direct action of the element present in the mandibular bone. One such mechanism could be the reduction in Zn concentration within mandibular bone tissue observed in the Cd5 and Cd50 groups. At both Cd exposure levels, Zn concentration in mandibular bone tissue decreased to a similar degree. Given that Cd^2+^ can interfere with Zn^2+^ during membrane transport, including absorption, exposure to this xenobiotic may contribute to Zn deficiency in the organism [24,46]. Consistent with this, we previously reported that rats exposed to Cd at 5 and 50 mg/L in drinking water exhibited lower serum Zn concentrations and lower femoral Zn content compared to the control group [23,24]. The serum Zn concentration is the leading indicator of the body’s status of this bioelement, and about 85% of the total amount of this element is found in the skeleton and muscular tissues. As in the present study, a simultaneous reduction in Zn concentration and SOD activity has already been reported in bone tissue collected from the distal femur [23], which has a structure similar to that of the mandibular bone [17]. Considering Zn’s role in modulating SOD activity and defending against oxidative stress, the diminished Zn concentration in the mandibular bone tissue observed in both Cd exposure groups may partially explain the absence of significant differences in markers of oxidative–reductive status between the moderate and relatively high exposure. Nonetheless, the numerous correlations noted between Cd concentration in mandibular bone and various indices of oxidative–reductive balance and markers of oxidative damage to cellular macromolecules (OGM, PC, and LPO) suggest that Cd’s pro-oxidative impact is contingent upon its concentration within the bone tissue and may intensify as its concentration increases. These correlations can be attributed to the antagonistic relationship between Cd and Zn in the tissue. As the concentration of Zn decreases with rising Cd accumulation, the antioxidative capacity of the bone correspondingly diminishes. Based on these findings, it is evident that while Cd’s pro-oxidative influence in the mandibular bone involves indirect pathways, it also critically depends on the concentration of this toxic element in the bone tissue.

The results of our previous study indicate that prolonged exposure to Cd at 5 and 50 mg/L, as well as low-level exposure at 1 mg/kg diet, disrupts the redox balance and induces oxidative damage to DNA, lipids, and proteins in long bones [23,25]. Al Ibrahim et al. [26] demonstrated that in the femurs of rats administered with Cd subcutaneously at a dose of 3 mg/kg b.w. over 12 weeks, there was a reduction in the activities of CAT, SOD, and GPx and an increase in PC and LPO concentrations. Yang et al. [49] noted decreased activities of CAT, SOD, and GPx and elevated malondialdehyde (MDA) concentration in the femoral bone of mice exposed to Cd at 50 mg/L for 12 weeks. Yin et al. [32] reported decreased activities of SOD and GPx, accompanied by elevated concentrations of markers of DNA damage, such as γ-H2A histone family member X (γ-H2AX) and 8-oxoguanine, in the mandibles of *Bmi1* gene knockout (Bmi1-/-) mice. *Bmi1* plays a crucial role in promoting dentin and alveolar process development in the mandible by regulating redox homeostasis. Given that redox homeostasis within the organs of the oral cavity can be compromised by numerous factors, including Cd [32,50], research aimed at elucidating whether Zn supplementation protects mandibular bone is of paramount importance.

The observation from the current study that nearly all measured markers of oxidative–reductive status in animals supplemented with Zn during Cd exposure (except for the lower SOD activity in the Cd5 + Zn30 group and TOS in the Cd50 + Zn30 and Cd50 + Zn60 groups) were not adversely affected compared to the control group suggests a beneficial effect of increased Zn intake under moderate and relatively high Cd exposure. Furthermore, the fact that concentrations of H_2_O_2_, OGM, and LPO in the Cd5 + Zn30 group reached even lower values, and TAS in the Cd50 + Zn30 and Cd50 + Zn60 groups was higher than in the control group, also indicates the advantageous impact of Zn supplementation in Cd-treated rats. It is noteworthy that, although in animals supplemented with Zn during exposure to Cd at 50 mg/L, TOS was significantly elevated compared to the control group (yet lower than in the Cd50 group), the administration of Zn augmented the antioxidative capacity of the mandibular bone beyond control levels, and this served to prevent disruption in the oxidative–reductive balance. The finding that the administration of Zn alone decreased the concentrations of H_2_O_2_ and OGM, and, at the higher level of supplementation, also reduced LPO concentration in mandibular bone, underscores the potent antioxidative properties of this bioelement [35,36]. Considering Zn’s robust antioxidative activity [35,36] and our data demonstrating its protective effect against Cd-induced oxidative stress across various tissues and organs [23,29,30,40,41], it is not surprising that Zn also mitigates the pro-oxidative effects of Cd in mandibular bone. Nevertheless, it was challenging to forecast that protection would remain effective even at relatively high exposure levels. The fact that Zn supplementation at 30 mg/L effectively mitigated the Cd-induced pro-oxidative effects at both exposure levels, with no discernible difference between the Cd5 + Zn30 and Cd5 + Zn60 groups, nor between the Cd50 + Zn30 and Cd50 + Zn60 groups, demonstrates that a 71% increase in the daily intake of Zn suffices to decrease Cd concentration and prevent its pro-oxidative effects in the mandibular bone.

Oxidative modifications of nucleic acids constitute some of the most significant effects of Cd-induced oxidative stress. These modifications underpin the carcinogenic potential of this element and may result in serious health consequences [3,51]. Increased lipid peroxidation compromises the structural integrity of biological membranes, including lysosomal membranes, leading to the release of hydrolytic enzymes into the cell and ultimately causing cell destruction [52]. An elevation in PC concentration within bone tissue results from oxidative alterations in the structure of bone proteins, consequently diminishing their biological activity [25]. Given that disruption of the redox balance within cells and tissues, including bone tissue, can have severe repercussions for their proper functioning, the present study’s finding that Zn supplementation can effectively mitigate this effect in mandibular bone underscores the role of this trace metal as a potent protective agent against the adverse impacts of Cd on the oral cavity.

Considering the results of our previous research, as well as those of other authors [23,24,25,30,40,41], it can be concluded that the protective effect of Zn on the mandibular bone, under Cd exposure, can be explained by the normalisation of the body’ bioelement status, including its concentration in the mandibular bone, as well as through the direct antioxidative action of this bioelement and its interaction with Cd. Cd^2+^ and Zn^2+^ compete during absorption in the gastrointestinal tract for the same transport proteins [2]. Consequently, increased Zn intake not only improves the body’s status of this bioelement but also may reduce Cd absorption from the intestines, thereby decreasing its accumulation in the body and counteracting the toxic effects of this xenobiotic. The protective role of Zn against the toxic effects of Cd may also be linked to its capacity to induce MT. This low-molecular-weight protein, which plays a key role in maintaining Zn homeostasis, is also crucial for sequestering Cd and mitigating its toxicity. Although both elements stimulate MT synthesis, Cd exhibits a higher binding affinity for this protein than Zn. Zn binding to MT results in the formation of stable complexes that chelate Cd^2+^, thereby detoxifying them [3,37,39,42,46].

In the current study, Cd concentrations in the mandibular bone tissue of rats administered Zn during exposure to this toxic element at concentrations of 5 and 50 mg/L were lower than those observed in animals not receiving Zn supplementation. Concurrently, the supplementation with Zn normalised the concentration of this essential element in the mandibular bone. The negative correlation between Cd and Zn concentrations in the mandibular bone, along with the observed relationships between these elements’ concentrations and the assessed indices of redox balance, indicates that the detrimental effects of Cd, including reduced Zn concentration and disturbance of redox homeostasis, escalate with increasing mandibular Cd concentration.

In view of the existing literature, the work presented herein is the inaugural study to assess and demonstrate the protective effect of increased Zn intake against oxidative-reductive imbalance in rat mandibular bone tissue during Cd exposure. The present study reveals that Zn supplementation protects against Cd-induced disruption of redox homeostasis in mandibular bone, thereby corroborating previous findings on Zn’s efficiency in safeguarding bone tissue from the detrimental effects of Cd. Both our results and those reported by other researchers suggest that Zn may play a pivotal role in defending bone tissue from the toxic impacts of Cd [23,24,52]. The protective influence of Zn on bone is multifaceted. This bioelement stimulates bone growth by activating enzymes that facilitate the synthesis of DNA, RNA, and proteins, enhances osteoblast activity, and promotes collagen synthesis. Conversely, it inhibits osteoclastic bone resorption, thus increasing bone turnover in favour of bone formation [52,53]. The results of the present study, taken together with prior findings in this same animal model [23,24], indicate that Zn’s protective properties are also related to its antioxidative capacity.

We acknowledge both the novelty and limitations of our research. The originality and innovation of our study lie in being the inaugural assessment of whether increased Zn intake can safeguard against the detrimental effects of chronic Cd exposure on mandibular bone, conducted in an experimental animal model simulating potential human exposure. Consequently, although with appropriate caution, these findings may be extrapolated to humans. The outcomes of this investigation may serve as a foundational reference for future research on the potential of Zn supplementation to reduce health risks associated with Cd exposure, including mandibular bone deterioration. However, this study also possesses certain limitations. One such limitation is that the effects of Cd and Zn were not examined at various time points throughout the 12-month study. This would have allowed the assessment of the progression of Cd-induced changes over time, as well as the accumulation of this xenobiotic in the mandibular bone, and thereby a more accurate evaluation of the relationship between Cd concentration in the bone and its oxidative-reductive status. Furthermore, such an approach would have permitted the assessment of the protective effect of Zn, depending on the duration of administration and Cd concentration in the mandibular bone. Regrettably, the current investigation did not include the quantification of non-enzymatic antioxidants’ concentrations, particularly GSH. Nonetheless, the TAS of the mandibular bone was evaluated, and previous research documented reduced GSH concentration in bone tissue at the distal femur [23]. A more comprehensive examination, considering lower Cd exposure levels that reflect the low exposure of the general population and assessing whether lower Zn supplementation is effective in protecting against Cd-induced effects on mandibular bone, would be advisable. Such investigations may be performed in the future. This investigation represents the first on this subject, and we acknowledge that further research is required to elucidate the involvement of Cd in the aetiology of mandibular bone damage and periodontal diseases, as well as the potential of Zn to mitigate these effects.

## 5. Conclusions

Our findings from the experimental animal study indicate that oral exposure to Cd, corresponding to moderate human exposure, disrupts the redox balance of mandibular bone tissue, resulting in oxidative stress and oxidative modifications of nucleic acids, proteins, and lipids. Furthermore, Zn supplementation has been shown to protect against these outcomes. These findings carry significant implications for the mandibular bone in humans. They appear to substantiate the hypothesis that Cd contributes to the development of oxidative stress in humans, while also emphasising the substantial protective role of Zn. The demonstration, within an experimental model of moderate and relatively high human exposure to Cd, that Zn supplementation exerts a protective effect on mandibular bone disorders caused by this toxic metal, attributable to its capacity to prevent oxidative stress and its sequelae, constitutes the most important and practically significant conclusion of this study. Indeed, this research may provide a foundation for further investigations into the use of Zn preparations to mitigate health risks in individuals exposed to Cd.

## Figures and Tables

**Table 1:** The intake of cadmium (Cd) and zinc (Zn) through drinking water in specific experimental groups [24].

Experimental Groups	Cd Intake [mg/kg b.w./24 h]	Zn Intake [mg/kg b.w./24 h]
Control	0	0
Zn30	0	0.982–3.910 ^1^
Zn60	0	2.260–7.710
Cd5	0.190–0.750 ^1^	0
Cd5 + Zn30	0.163–0.745	0.980–3.950
Cd5 + Zn60	0.175–0.753	2.200–6.820
Cd50	1.740–4.340	0
Cd50 + Zn30	1.810–4.370	0.997–3.980
Cd50 + Zn60	1.800–4.440	2.020–7.520

^1^ Data are presented as the minimum and maximum intake within the specific experimental group.

**Table 2:** Spearman’s correlations between zinc (Zn) and cadmium (Cd) concentrations and markers of oxidative–reductive status of the mandibular bone.

Parameters		Markers of the Antioxidative Status					Markers of Oxidative Status					
		SOD	CAT	GPx	GR	TAS	OGM	PC	LPO	H_2_O_2_	TOS	OSI
**Markers of Antioxidative Status**												
	CAT	0.413 ^‡ 1^	-									
	GPx	0.696 ^‡^	0.397 ^‡^	-								
	GR	NS	−0.237 *	NS	-							
	TAS	0.728 ^‡^	0.750 ^‡^	0.641 ^‡^	NS	-						
**Markers of Oxidative Status**												
	OGM	NS	−0.474 ^‡^	NS	0.636 ^‡^	−0.318 ^†^	-					
	PC	NS	−0.504 ^‡^	−0.362 ^†^	0.375 ^†^	−0.429 ^‡^	0.694 ^‡^	-				
	LPO	NS	−0.390 ^‡^	NS	0.679 ^‡^	NS	0.914 ^‡^	0.717 ^‡^	-			
	H_2_O_2_	NS	−0.483 ^‡^	NS	0.653 ^‡^	−0.274 *	0.928 ^‡^	0.696 ^‡^	0.927 ^‡^	-		
	TOS	NS	NS	−0.416 ^‡^	0.522 ^‡^	NS	0.585 ^‡^	0.466 ^‡^	0.589 ^‡^	0.464 ^‡^	-	
	OSI	−0.328 ^†^	–0.342 ^‡^	–0.486 ^‡^	0.391 ^†^	−0.461 ^‡^	0.569 ^‡^	0.530 ^‡^	0.522 ^‡^	0.458 ^‡^	0.854 ^‡^	
Zn		0.275 *	0.418 ^‡^	0.530 ^‡^	−0.434 ^‡^	0.394 ^‡^	−0.458 ^‡^	−0.366 ^‡^	−0.461 ^‡^	−0.435 ^‡^	−0.475 ^‡^	−0.480 ^‡^
Cd		−0.255 *	NS	−0.483 ^‡^	0.452 ^‡^	NS	0.437 ^‡^	0.293 ^†^	0.415 ^‡^	0.302 ^‡^	0.722 ^‡^	0.627 ^‡^

^1^ Values represent the correlation coefficient r, where * *p* < 0.05; ^†^ *p* < 0.01; ^‡^ *p* < 0.001; NS, *p* > 0.05. CAT, catalase; GPx, glutathione peroxidase; GR, glutathione reductase; H_2_O_2_, hydrogen peroxide; LPO, lipid peroxides; OGM, oxidised guanine metabolites; OSI, oxidative stress index; PC, protein carbonyls; SOD, superoxide dismutase; TAS, total antioxidative status; TOS, total oxidative status.

**Table 3:** Spearman’s correlations between total antioxidative status (TAS), total oxidative status (TOS), oxidative stress index (OSI), and the concentrations of Cd and Zn in the mandibular bone, and these element concentrations in the blood/serum and their urinary excretion ^1^.

Parameters	Cd Concentration		Zn Concentration	
	Blood	24-h Urine	Serum	24-h Urine
TAS	NS	NS	NS	NS
TOS	0.643 ^‡ 2^	0.575 ^‡^	−0.325 ^†^	0.643 ^‡^
OSI	0.543 ^‡^	0.384 ^‡^	NS	0.534 ^‡^
Cd	0.874 ^‡^	0.806 ^‡^	−0.583 ^‡^	0.394 ^‡^
Zn	−0.355 ^†^	−0.240 *	NS	NS

^1^ Detailed data on Cd and Zn concentrations in the blood/serum and 24-h urine are reported [24] and summarised in Table S1 (Cd) and Table S3 (Zn). ^2^ Values represent the correlation coefficient (r), where * *p* < 0.05; ^†^ *p* < 0.01; ^‡^ *p* < 0.001; NS, *p* > 0.05.

## Data Availability

The original contributions presented in the study are included in the article/Supplementary Material; further inquiries can be directed to the corresponding author.

## Supplementary Materials

The following supporting information can be downloaded at: https://www.mdpi.com/article/10.3390/antiox14121480/s1, Table S1: The concentration of cadmium (Cd) in the blood and urine of rats in particular experimental groups; Table S2: Cadmium (Cd) concentration in the blood and urine of inhabitants of industrialised countries; Table S3: The concentrations of zinc (Zn) in the serum and 24-h urine in particular experimental groups; Table S4: The influence of cadmium (Cd) and/or zinc (Zn) on total antioxidative status (TAS), total oxidative status (TOS), and oxidative stress index (OSI) in the mandibular bone tissue; Table S5: The influence of cadmium (Cd) and/or zinc (Zn) on the activities of superoxide dismutase (SOD), catalase (CAT), glutathione peroxidase (GPx), and glutathione reductase (GR) in the mandibular bone tissue; Table S6: The influence of cadmium (Cd) and/or zinc (Zn) on the concentrations of oxidised guanine metabolites (OGM), protein carbonyls (PC), lipid peroxides (LPO) and hydrogen peroxide H_2_O_2_ in the mandibular bone tissue; Table S7: The influence of cadmium (Cd) and/or zinc (Zn) on the concentrations of these metals in the mandibular bone tissue. References [54,55,56,57,58,59,60,61,62,63,64,65] are cited in the Supplementary Material.

## Abbreviations

8-OHdG	8-hydroxy-2′-deoxyguanosine
8-OHG	8-hydroxyguanosine
b.w.	body weight
CAT	catalase
Cd	cadmium
Cd^2+^	cadmium ion
CdCl_2_	cadmium chloride
Cu	copper
Cu^+^	copper(I) ion
CV	coefficient of variation
DNA	deoxyribonucleic acid
DPPH	1,1-diphenyl-2-picrylhydrazyl
ELISA	enzyme-linked immunosorbent assay
Fe	iron
Fe^2+^	iron(II) ion
GPx	glutathione peroxidase
GR	glutathione reductase
GSH	reduced glutathione
H_2_O_2_	hydrogen peroxide
LPO	lipid peroxides
Mn	manganese
MDA	malondialdehyde
MT	metallothionein
-OH	hydroxyl group
OGM	oxidised guanine metabolites
OSI	oxidative stress index
*p*	level of statistical significance
PC	protein carbonyl groups
r	correlation coefficient
RNA	ribonucleic acid
ROS	reactive oxygen species
Se	selenium
SE	standard error
Se^2+^	selenium ion
-SH	sulfhydryl groups
SOD	superoxide dismutase
TAS	total antioxidative status
TOS	total oxidative status
Zn	zinc
Zn^2+^	zinc ion
ZnCl_2_	zinc chloride
γ-H2AX	γ-H2A histone family member X
