# Supplementary material for: The Protective Effect of Zinc Supplementation Against Oxidative Stress and Oxidative Modifications of Cellular Macromolecules in the Mandibular Bone of Rats Exposed to Cadmium
Source: Antioxidants (Basel). 2025 Dec 10;14(12):1480. doi: 10.3390/antiox14121480 (PMC12729647; doi:10.3390/antiox14121480)
Supplement: Supplementary file 1 [file antioxidants-14-01480-s001.zip › antioxidants-3913712-supplementary.pdf]

Supplementary Materials

The Protective Effect of Zinc Supplementation against Oxidative Stress and Oxidative Modifications of Cellular Macromolecules in the Mandibular Bone of Rats Exposed to Cadmium

Kamil Bijowski[1,*], Ewa Dąbrowska[2], Małgorzata M. Brzóska[3], Joanna Rogalska[3], Karolina Orywal[4], Zofia N. Dąbrowska[5] and Jan Borys[1]

[1] Department of Maxillofacial and Plastic Surgery, Medical University of Bialystok; M. Skłodowskiej-Curie 24A Street, 15-276 Białystok, Poland; kamil.bijowski@umb.edu.pl

[2] Independent Gerostomatology Laboratory, Medical University of Bialystok, Akademicka 3 Street, 15-267 Białystok, Poland;

[3] Department of Toxicology, Medical University of Bialystok, A. Mickiewicza 2C Street, 15-222 Bialystok, Poland;

[4] Department of Biochemical Diagnostics, Medical University of Bialystok, J. Waszyngtona 15A Street, 15-269 Białystok, Poland

[5] Department of Radiology, Medical University of Bialystok, M. Skłodowskiej-Curie 24A Street, 15-276 Białystok

* Correspondence: kamil.bijowski@umb.edu.pl

Table S1. The concentration of cadmium (Cd) in the blood and urine of rats in particular experimental groups. [1]

Experimental Group	Cd in the Blood (μg/L)	Cd in the Urine (μg/24 h)
Control	0.319 ± 0.021	0.0080 ± 0.0006
	0.244 – 0.400 [2]	0.0054 – 0.0102
Zn30	0.322 ± 0.027	0.0061 ± 0.0012
	0.210 – 0.445	0.0037 – 0.0141
	↔	↔
Zn60	0.362 ± 0.028	0.0064 ± 0.0010
	0.260 – 0.476	0.0044 – 0.0133
	↔	↔
Cd5	1.615 ± 0.139	0.0140 ± 0.0021
	1.223 – 2.121	0.0078 – 0.0262
	↑ 5-fold	↑ 1.8-fold
Cd5 + Zn30	1.724 ± 0.107	0.0197 ± 0.0023
	1.104 – 2.068	0.0085 – 0.0291
	↑ 5.4-fold	↑ 2.5-fold
Cd5 + Zn60	1.746 ± 0.123	0.0138 ± 0.0015
	1.340 – 2.418	0.0061 – 0.0191
	↑ 5.5-fold	↑ 1.7-fold
Cd50	16.09 ± 0.301	0.2719 ± 0.0223
	14.97 – 17.20	0.2027 – 0.3647
	↑ 50-fold	↑ 34-fold
Cd50 + Zn30	14.00 ± 0.397	0.2753 ± 0.0184
	12.19 – 15.72	0.1848 – 0.3415
	↑ 44-fold; ↘ *13%*	↑ 34-fold
Cd50 + Zn60	14.14 ± 0.490	0.3512 ± 0.0463
	11.21 – 15.60	0.2051 – 0.5769
	↑ 44-fold; ↘ *12%*	↑ 44-fold

[1] Detailed data on Cd concentration in all experimental groups are reported [Brzóska, M.M.; Rogalska, J.; Gałażyn-Sidorczuk, M.; Jurczuk, M.; Roszczenko, A.; Kulikowska-Karpińska, E.; Moniusz-ko-Jakoniuk, J. Effect of zinc supplementation on bone metabolism in male rats chronically exposed to cadmium. *Toxicology* **2007**, *237*, 89–103. https://doi.org/10.1016/j.tox.2007.05.001]. [2] Values represent the mean ± standard error (SE) and range of concentrations (minimum – maximum) for eight rats in each experimental group. ↑ and ↔, higher (fold of change or percentage change) or lack of change, respectively, compared to the control group; ↘, lower than in the Cd50 group.

Table S2. Cadmium (Cd) concentration in the blood and urine of inhabitants of industrialised countries.

<table>
<tr><th rowspan="2">Country</th><th rowspan="2">Form of the Expression of Cd Concentration</th><th colspan="2">Blood (µg/L)</th><th colspan="2">Urine (µg/g creatinine)</th><th rowspan="2">Reference</th></tr>
<tr><th>Female</th><th>Male</th><th>Female</th><th>Male</th></tr>
<tr><td>Brazilian population</td><td>Mean ± SD
Median
Min – Max</td><td colspan="2">0.70 ± 0.20
0.69
0.28 – 1.38</td><td colspan="2">0.61 ± 0.65
0.38
0.09 – 3.63</td><td>Perini et al. 2024</td></tr>
<tr><td>China</td><td>Mean ± SD</td><td>0.43 ± 0.051</td><td>0.51 ± 0.055</td><td></td><td></td><td>Lei et al. 2024</td></tr>
<tr><td>Brazil</td><td>Median
P25, P75</td><td colspan="2"></td><td colspan="2">0.41
0.19, 0.92</td><td>Coutinho et al. 2023</td></tr>
<tr><td>Pakistan
Mining workers
Textile woollen mill workers</td><td>
Mean ± SD
Mean ± SD</td><td colspan="2">
3.89 ± 0.46
5.75 ± 0.11</td><td colspan="2"></td><td>Khan et al. 2023</td></tr>
<tr><td>French population (environmental and dietary exposure)</td><td>
GM
Min – Max</td><td colspan="2"></td><td colspan="2">
0.23
0.21 – 0.26</td><td>Jeanjean et al. 2022</td></tr>
<tr><td>Southern Korea</td><td>95% CI
Min – Max</td><td>1.01
0.99 – 1.03</td><td>0.76
0.74 – 0.77</td><td></td><td></td><td>Park et al. 2021</td></tr>
<tr><td>Sweden</td><td>Mean
Median</td><td>0.43
0.29</td><td>0.32
0.19</td><td></td><td></td><td>Barregard et al. 2021</td></tr>
<tr><td>Sweden</td><td>Mean
Median</td><td colspan="2"></td><td colspan="2">0.31
0.24</td><td>Wallin et al. 2021</td></tr>
<tr><td>Brasilian winegrowers</td><td>Mean
Min – Max</td><td>0.47
<LOQ – 9.43</td><td>0.30
<LOQ – 11.38</td><td>0.38
<LOQ – 8.16</td><td>0.26
<LOQ – 5.60</td><td>Lini et al. 2021</td></tr>
<tr><td>Brazilian foundry workers</td><td>Mean ± SD
Min – Max</td><td colspan="2">1.19 ± 1.14
0.51 – 5.06</td><td colspan="2">0.17 ± 0.10
0.05 – 0.65</td><td>Freire et al. 2021</td></tr>
<tr><td>China</td><td>Mean ± SD
Min – Max
Median
5 - 95th P</td><td colspan="2">0.98 ± 0.37
0.42 – 1.92
0.91
0.43 – 1.65</td><td colspan="2">0.38 ± 0.30 µg/L
0.12 – 1.67 µg/L
0.25 µg/L
0.14 – 1.15 µg/L</td><td>Liu et al. 2021</td></tr>
<tr><td>Senegal (environmental exposure)</td><td>Mean ± SD</td><td colspan="2">1.78 ± 0.70</td><td colspan="2">1.25 ± 0.90</td><td>Cabral et al. 2021</td></tr>
<tr><td>Northern Sweden (environmental and dietary exposure)</td><td>
Mean ± SD
Median
Min – Max
5 - 95th P</td><td>
0.37 ± 0.42
0.29
0.05 – 5.7
0.14 – 0.77</td><td></td><td>µg/L
0.13 ± 0.10
0.10
0.02 – 1.1
0.04 – 0.27</td><td></td><td>Gustin et al. 2020</td></tr>
<tr><td>U.S.A. (tobacco smokers)</td><td>GM
95% CI</td><td colspan="2">1.17
0.77 – 1.81</td><td colspan="2"></td><td>Domingo-Relloso et al. 2020</td></tr>
<tr><td>U.S.A. (tobacco smokers)</td><td>UGM
95% CI</td><td colspan="2">8.0
7.5 – 8.6</td><td colspan="2"></td><td>Jain 2019</td></tr>
</table>

GM, geometric mean; 95% CI, 95% confidence interval; IQR, interquartile range; Min, minimal value; Max, maximal value; SD, standard deviation; UGM, unadjusted geometric mean; 5-95th P, 5-95th percentiles.

References

Perini, J.A.; Silva, Y.M.H.D.; Silva, M.C.D.; Silva, B.P.; Machado, D.E.; Moreira, M.F.R. Cadmium exposure and noncommunicable diseases in environmentally exposed Brazilian population: cross-sectional study without association of GSTP1 polymorphism. *Toxics* **2024**, *12*, 640. https://doi.org/10.3390/toxics12090640.

Lei, Y.; Guo, M.; Xie, J.; Liu, X.; Li, X.; Wang, H.; Xu, Y.; Zheng, D. Relationship between blood cadmium levels and bone mineral density in adults: a cross-sectional study. *Front. Endocrinol.* **2024,** *15*, 1354577. https://doi.org/10.3389/fendo.2024.1354577.

Coutinho, G.B.F.; Moreira, M.d.F.R.; Fischer, F.M.; dos Santos, M.C.R.; Feitosa, L.F.; de Azevedo, S.V.; Borges, R.M.; Nasciento-Sales, M.; Christoffolete, M.A.; SantaMarinha, M.S.; et al. Influence of environmental exposure to steel waste on endocrine dysregulation and per3 gene polymorphisms. *Int. J. Environ. Res. Public Health* **2023**, *20*, 4760. https://doi.org/10.3390/ijerph20064760.

Khan, K.; Rafiq, M.T.; Bacha, A.U.; Nabi, I.; Irshad, M.; Faridullah, F.; Younas, M.; Khan, M.D.; Aziz, R.; Amin, M.; Arifeen, A.; Aslam, S.; Ahmad, S.; Iqbal, A. Assessment of heavy metals and associated oxidative stress in occupationally exposed workers from Bannu and Karak Districts in Pakistan. *Environ Geochem Health***. 2023**, *8*, 5915–5925. https://doi.org/10.1007/s10653-023-01603-x.

Jeanjean, M.; Goix, S.; Dron, J.; Periot, M.; Austruy, A.; Douib, K.; Persoons, R.; Etienne, M.P.; Revenko, G.; Chamaret, P. Influence of environmental and dietary exposures on metals accumulation among the residents of a major industrial harbour (Fos-sur-Mer, France*). J. Trace Elem. Med. Biol.* **2022**, *73*, 127021. https://doi.org/10.1016/j.jtemb.2022.127021.

Park, E.; Kim, J.; Kim, B.; Park, E.Y. Association between environmental exposure to cadmium and risk of suspected non-alcoholic fatty liver disease. *Chemosphere* **2021**, *246*, 128947. https://doi.org/10.1016/j.chemosphere.2020.128947.

Barregard, L.; Sallsten, G.; Harari, F.; Andersson, E. M.; Forsgard, N.; Hjelmgren, O.; Angerås, O.; Fadman, E.; Persson, M.; Lundh, T.; Borné, T.; Fagerbere, B.; Engström, G.; Bergström, G. Cadmium exposure and coronary artery atherosclerosis: A cross-sectional population-based study of Swedish middle-aged adults. *Environ. Health Perspect*. **2021**, *129*, 67007. https://doi.org/10.1289/EHP8523.

Wallin, M.; Barregard, L.; Sallsten, G.; Lundh, T.; Sundh, D.; Lorentzon, M.; Ohlsson, C.; Mellström, D. Low-level cadmium exposure is associated with decreased cortical thickness, cortical area and trabecular bone volume fraction in elderly men: The MrOS Sweden study. *Bone* **2021**, *43*, 115768. https://doi.org/10.1016/j.bone.2020.115768.

Lini, R.S.; Aguera, R.G.; Hoeltgebaum, D.; Paniz, F.P.; Pedron, T.; Capelari, S.; Monteiro, L.R.; Machinski Junior, M.; Nerilo, S.B.; Batista, B.L.; de Oliveira, M.L.F.; Mossini, S.A.G. Elemental plasma content and urinary excretion in vineyard farmers occupationally exposed to pesticides in southern Brazil. *Environ. Sci. Pollut. Res. Int.* **2021**, *28*, 51841–51853. https://doi.org/10.1007/s11356-021-14384-6.

Freire, B.M.; Gonzaga, R.G.; Pedron, T.; Monteiro, L.R.; Lange, C.N.; Pedreira Filho, W.D.R.; Batista, B.L. Occupational exposure to potentially toxic elements in the foundry industry: an integrated environmental and biological monitoring. *Environ. Sci. Pollut. Res. Int.* **2021**, *28*, 34630–34641. https://doi.org/10.1007/s11356-021-13099-y.

Liu, N.; Guan, Y.; Li, B.; Yao, S. Biomonitorization of concentrations of 28 elements in serum and urine among workers exposed to indium compounds. PLoS One **2021**, *16*, e0246943. https://doi.org/10.1371/journal.pone.0246943.

Cabral, M.; Garçon, G.; Touré, A.; Bah, F.; Dewaele, D.; Bouhsina, S.; Cazier, F.; Faye, A.; Fall, M.; Courcot, D.; Verdin, A. Renal impairment assessment on adults living nearby a landfill: Early kidney dysfunction biomarkers linked to the environmental exposure to heavy metals.*Toxicol. Rep*. **2021**, *8*, 386–394. https://doi.org/10.1016/j.toxrep.2021.02.009.

Gustin, K.; Barman, M.; Stråvik, M.; Levi, M.; Englund-Ögge, L.; Murray, F.; Jacobsson, B.; Sandberg, A.S.; Sandin, A.; Wold, A.E.; Vahter, M.; Kippler, M. Low-level maternal exposure to cadmium, lead, and mercury and birth outcomes in a Swedish prospective birth-cohort. *Environ. Pollut.* **2020**, *265* (Pt B), 114986. https://doi.org/10.1016/j.envpol.2020.114986.

Domingo-Relloso, A.; Riffo-Campos, A.L.; Haack, K.; Rentero-Garrido, P.; Ladd-Acosta, C.; Fallin, D.M.; Tang, W.Y.; Herreros-Martinez, M.; Gonzalez, J.R.; Bozack, A.K.; Cole, S.A.; Navas-Acien, A.; Tellez-Plaza, M. Cadmium, smoking, and human blood DNA methylation profiles in adults from the strong heart study. *Environ. Health Perspect.* **2020**, *128*, 1–14. https://doi.org/10.1289/EHP6345.

Jain, R.B. Concentrations of cadmium, lead, and mercury in the blood among US cigarettes, cigars, electronic cigarettes, and dual cigarette-e-cigarette users. *Environ. Pollut.* **2019**, *251*, 970–974. https://doi.org/10.1016/j.envpol.2019.05.041.

Table S3. The concentrations of zinc (Zn) in the serum and 24-h urine in particular experimental groups. [1]

Experimental Group	Zn in the Serum (μg/mL)	Zn in the Urine (μg/24 h)
Control	1.432 ± 0.068<br>1.255 – 1.795 [2]	2.776 ± 0.147<br>2.364 – 3.576
Zn30	1.498 ± 0.039<br>1.360 – 1.725<br>↔	3.198 ± 0.270<br>2.312 – 4.415<br>↔
Zn60	1.378 ± 0.048<br>1.255 – 1.580<br>↔	4.922 ± 0.474<br>2.641 – 6.650<br>↑ 77%
Cd5	1.208 ± 0.048<br>0.925 – 1.365<br>↓ 16%	2.752 ± 0.324<br>1.925 – 4.652<br>↔
Cd5 + Zn30	1.126 ± 0.045<br>0.920 – 1.305<br>↓ 21%	2.344 ± 0.190<br>1.903 – 3.160<br>↔
Cd5 + Zn60	1.193 ± 0.083<br>0.865 – 1.480<br>↓ 17%	3.503 ± 0.257<br>2.650 – 4.616<br>↑ 26%
Cd50	1.204 ± 0.081<br>0.860 – 1.645<br>↓ 16%	6.163 ± 0.400<br>4.381 – 7.399<br>↑ 2.2-fold
Cd50 + Zn30	1.066 ± 0.045<br>0.835 – 1.215<br>↓ 26%	6.099 ± 0.405<br>4.290 – 7.884<br>↑ 2.2-fold
Cd50 + Zn60	1.043 ± 0.038<br>0.865 – 1.170<br>↓ 27%	5.489 ± 0.289<br>4.082 – 6.770<br>↑ 2-fold

[1] Detailed data on Zn concentration in all experimental groups are reported [Brzóska, M.M.; Rogalska, J.; Gałażyn-Sidorczuk, M.; Jurczuk, M.; Roszczenko, A.; Kulikowska-Karpińska, E.; Moniusz-ko-Jakoniuk, J. Effect of zinc supplementation on bone metabolism in male rats chronically exposed to cadmium. *Toxicology* **2007**, 237, 89–103. https://doi.org/10.1016/j.tox.2007.05.001]. [2] Values represent the mean ± standard error (SE) and range of concentrations (minimum – maximum) for eight rats in each experimental group. ↑, ↓, and ↔, higher, lower (fold of change or percentage change) or lack of change, respectively, compared to the control group.

Table S4. The influence of cadmium (Cd) and/or zinc (Zn) on total antioxidative status (TAS), total oxidative status (TOS), and oxidative stress index (OSI) in the mandibular bone tissue.

Group	TAS (nmol/mg protein)	TOS (nmol/mg protein)	OSI
Control	428.5	3.252	0.0079
	368.4 – 504.3	2.040 – 5.901	0.0054 – 0.0129
Zn30	493.5	4.134	0.0082
	467.7 – 622.5	2.541 – 5.347	0.0058 – 0.0101
Zn60	498.4	3.123	0.0071
	322.6 – 521.6	1.704 – 4.379	0.0036 – 0.0140
Cd5	242.3 $a^‡$ $b^‡$ $c^‡$	11.68 $a^‡$ b^* $c^†$	0.0462 $a^‡$ $b^‡$ $c^‡$
	165.3 – 362.0	10.24 – 13.67	0.0237 – 0.0927
Cd5 + Zn30	401.6 $d^†$	3.066 $d^†$	0.0042 $d^‡$
	307.8 – 531.3	2.805 – 5.440	0.0035 – 0.0171
Cd5 + Zn60	485.8 $d^†$	4.692 d^*	0.0123 $d^‡$
	329.2 – 571.4	3.614 – 7.423	0.0042 – 0.0147
Cd50	180.8 $a^‡$ $b^‡$ $c^‡$ f^*	10.08 $a^‡$ $b^*c^†$ $e^†$	0.0573 $a^‡$ $b^‡$ $c^‡$ $e^‡$
	151.6 – 269.9	8.541 – 16.34	0.0307 – 0 0915
Cd50 + Zn30	646.0 a^* $d^‡$ $g^‡$	6.742 $a^‡$ c^* $e^†$ g^*	0.0115 $g^‡$
	403.2 – 742.3	6.058 – 10.32	0.0082 – 0.0250
Cd50 + Zn60	660.3 $a^†$ $d^‡$ $g^‡$	8.238 $a^‡$ c^* $e^†$ g^*	0.0117 $g^‡$
	508.0 – 910.6	4.251 – 10.92	0.0067 – 0.0203
Effect size (η^2)	0.657	0.753	0.581

Values represent the median and range of concentrations (minimum – maximum) for 8 animals per group. * $p < 0.05$, † $p < 0.01$, and ‡ $p < 0.001$ relative to a – control group, b – Zn30 group, c – Zn60 group, d – Cd5 group, e – Cd5 + Zn30 group, f – Cd5 + Zn60 group, and g – Cd50 group.

Table S5. The influence of cadmium (Cd) and/or zinc (Zn) on the activities of superoxide dismutase (SOD), catalase (CAT), glutathione peroxidase (GPx), and glutathione reductase (GR) in the mandibular bone tissue.

Group	SOD (U/mg protein)	CAT (mmol H_2O_2/min/ mg protein)	GPx (mU/mg protein)	GR (mU/mg protein)
Control	10.02	4.098	175.9	13.79
	7.670 – 11.62	3.475 – 7.489	79.88 – 224.7	10.79 – 28.39
Zn30	8.726	7.514	159.2	15.10
	7.673 – 10.06	6.302 – 10.95	131.7 – 199.3	11.96 – 32.27
Zn60	8.822	6.563	158.3	8.487 a*
	7.812 – 10.61	5.529 – 9.606	140.6 – 179.6	5.310 – 10.14
Cd5	6.711 a*	3.822	92.09 a‡ b* c*	18.60 c‡
	6.149 – 7.556	3.132 – 5.621	70.80 – 100.9	17.25 – 20.46
Cd5 + Zn30	5.911 a†	14.58 a† d†	97.93	6.890 a† d‡
	5.022 – 7.726	9.055 – 17.34	84.30 – 122.5	4.812 – 10.75
Cd5 + Zn60	9.193 d*	16.03 a† d†	126.6 d*	6.554 a† d‡
	6.110 -10.78	14.49 – 19.64	102.8 – 183.0	5.377 – 8.577
Cd50	5.380 a†	1.506 a* e† f‡	57.14 a† b‡ c‡	20.16 c‡ e‡ f‡
	4.435 – 9.427	1.147 – 2.692	37.90 – 78.60	11.91 – 25.91
Cd50 + Zn30	9.786 g†	16.35 a† d† g‡	121.2 g†	17.39 c* e† f*
	6.230 – 10.57	11.65 – 19.08	87.50 – 164.4	13.33 – 20.00
Cd50 + Zn60	9.763 e† g‡	18.57 a‡ d‡ g‡	144.8 g‡	16.88 c* e† f*
	8.446 – 13.96	17.05 – 20.60	141.3 – 188.7	14.43 – 18.64
Effect size (η2)	0.500	0.890	0.640	0.684

Values represent the median and range of concentrations (minimum – maximum) for 8 animals per group. * $p < 0.05$, † $p < 0.01$, and ‡ $p < 0.001$ relative to a – control group, b – Zn30 group, c – Zn60 group, d – Cd5 group, e – Cd5 + Zn30 group, f – Cd5 + Zn60 group, and g – Cd50 group.

Table S6. The influence of cadmium (Cd) and/or zinc (Zn) on the concentrations of oxidised guanine metabolites (OGM), protein carbonyls (PC), lipid peroxides (LPO), and hydrogen peroxide H_2O_2 in the mandibular bone tissue.

Group	OGM (pg/mg protein)	PC (ng/mg protein)	LPO (µM/g protein)	H_2O_2 (nmol/mg protein)
Control	399.2	31.30	4.973	128.6
	331.6 – 446.3	25.86 – 46.30	4.131 – 6.480	105.9 – 150.4
Zn30	287.4 a*	32.84	4.293	91.27 a*
	248.9 – 353.4	24.62 – 42.69	3.685 – 7.093	76.52 – 157.8
Zn60	277.3 a*	32.29	4.010 a*	86.83 a†
	211.5 – 346.2	24.28 – 42.17	2.886 – 5.071	64.37 – 106.1
Cd5	464.0 a* c‡	111.2 a‡ b‡ c‡	8.100 a‡ b† c‡	141.3 c†
	448.8 – 602.8	87.97 – 129.0	7.781 – 12.06	136.7 – 182.7
Cd5 + Zn30	199.1 a‡ d‡	23.25 d‡	2.599 a† d‡	60.85 a‡ d‡
	168.6 – 294.3	20.61 – 38.05	2.307 – 4.687	51.44 – 87.23
Cd5 + Zn60	332.4 d*	35.08 d†	4.832 d‡	101.7 d†
	245.2 – 390.1	32.38 – 45.42	3.737 – 5.672	76.23 – 116.7
Cd50	464.6 a*c† e‡	107.5 a‡ b‡ c‡ e‡	7.206 a† b* c‡ e‡	273.8 a† b*c‡ e‡ f*
	420.3 – 561.0	103.2 – 123.1	5.884 – 12.81	201.8 – 306.7
Cd50 + Zn30	325.2 g†	27.09 d‡ f† g‡	4.668 d† g‡	273.8 a† b*c‡ e‡ f*
	240.8 – 373.8	21.98 – 30.32	3.350 – 5.821	201.8 – 306.7
Cd50 + Zn60	303.6 g†	30.07 d† g‡	4.479 d† g†	92.37 g†
	275.2 – 399.4	22.19 – 35.65	3.691 – 6.011	83.60 – 108.1
Effect size (η^2)	0.755	0.631	0.646	0.747

Values represent the median and range of concentrations (minimum – maximum) for 8 animals per group. * $p < 0.05$, † $p < 0.01$, and ‡ $p < 0.001$ relative to a – control group, b – Zn30 group, c – Zn60 group, d – Cd5 group, e – Cd5 + Zn30 group, f – Cd5 + Zn60 group, and g – Cd50 group.

Table S7. The influence of cadmium (Cd) and/or zinc (Zn) on the concentrations of these metals in the mandibular bone tissue.

Group	Zn (μg/g)	Cd (μg/g)
Control	276.3	0.0094
	205.3 – 302.1	0.0058 – 0.0130
Zn30	280.7	0.0094
	218.8 – 353.8	0.0072 – 0.0110
Zn60	288.3	0.0084
	252.1 – 320.7	0.0054 – 0.0251
Cd5	197.7 a*	0.1441 a† b† c†
	174.5 – 286.1	0.1203 – 0.2825
Cd5 + Zn30	271.5 d†	0.0505 a* b* c* d*
	254.1 – 338.3	0.0401 – 0.0722
Cd5 + Zn60	275.0 d†	0.0608 a* b* c* d*
	256.5 – 307.7	0.0453 – 0.0886
Cd50	200.5 a* e† f†	0.3795 a‡ b‡ c‡ d‡ e† f‡
	182.9 – 252.9	0.2463 – 0.5309
Cd50 + Zn30	266.3 d* g†	0.2088 a‡ b‡ c‡ e* f* g†
	242.5 – 285.8	0.1101 – 0.2463
Cd50 + Zn60	263.6 d* g†	0.2443 a‡ b‡ c‡ d† e† f† g†
	252.1 – 297.6	0.2000 – 0.3612
Effect size (η^2)	0.914	0.362

Values represent the median and range of concentrations (minimum – maximum) for 8 animals per group. * $p < 0.05$, $^{\dagger}p < 0.01$, and $^{\ddagger}p < 0.001$ relative to a – control group, b – Zn30, c – Zn60, d – Cd5 group, e – Cd5 + Zn30 group, f – Cd5 + Zn60 group, and g – Cd50.
